# “Space to see the future”? A political economy analysis of child and adolescent mental health and well-being in Ethiopia including routes for change

**DOI:** 10.3389/fsoc.2024.1488619

**Published:** 2025-02-06

**Authors:** Kibur Engdawork, Lucia D’Ambruoso, Tsion Hailu, Mahlet Yared, Girma M. Geletu, Semere G. Baraki, Elias Sebsibe, Pamela Abbott

**Affiliations:** ^1^College of Social Sciences, Addis Ababa University, Addis Ababa, Ethiopia; ^2^College of Health Sciences, Addis Ababa University, Addis Ababa, Ethiopia; ^3^Centre for Global Development, School of Education, University of Aberdeen, Aberdeen, United Kingdom; ^4^Aberdeen Centre for Health Data Science, School of Medicine, Medical Sciences and Nutrition, University of Aberdeen, Aberdeen, United Kingdom; ^5^Department of Epidemiology and Global Health, Umeå University, Umeå, Sweden; ^6^MRC/Wits Rural Public Health and Health Transitions Research Unit (Agincourt), School of Public Health, University of the Witwatersrand, Johannesburg, South Africa; ^7^Department of Global Health, Faculty of Medicine and Health Sciences, Stellenbosch University, Stellenbosch, South Africa; ^8^Public Health, National Health Service (NHS) Grampian, Aberdeen, United Kingdom; ^9^Dilchora Referral Hospital, Dire Dawa, Ethiopia

**Keywords:** child and adolescent mental health, child and adolescent mental well-being, Ethiopia, political economy analysis, political economy

## Abstract

**Background:**

Ethiopia faces significant mental health challenges; mental disorders are the leading non-communicable condition, and many adults experience symptoms by age 14–15. We examined structural, institutional, and agentic factors affecting child and adolescent mental well-being in Ethiopia. The aims were to describe the political, economic, social and policy contexts in which mental well-being exists; analysing power, interests, and the influence and resources key stakeholders bring to bear on decision-making processes related to child and adolescent well-being.

**Methods:**

We used a Political Economy Analysis framework to identify structural and institutional features, networks of influence, and routes for change. Using this lens, a literature review was performed, supplemented with key informant interviews (*n* = 9).

**Results:**

Multiple structural drivers of mental health problems were identified: globalized urbanization and social fragmentation, and political and economic systems characterized by economic ambition but entrenched poverty, aid dependency, conflict and civil war. Despite significant policy recognition and support, there was poor coordination between federal and regional structures and sectors, vastly insufficient resources, and low coverage especially in rural areas. Service delivery was further complicated by widespread and normalised violence against children and young people, low knowledge and awareness among children and families, entrenched stigma and gender inequities, and ethnic tensions, conflict and displacement. Overall, regional resource shortages drastically limited the collective agencies of service users and providers. Meanwhile, development programmes lack context and coordination. The analysis emphasizes an urgent need to enhance implementation support through targeted, contextually relevant mental health policies and programmes, and institutional expansion of spaces and processes for multisectoral working, especially between health and education. Development partners should align with statutory bodies as a mechanism to harmonise and contextualise.

**Conclusion:**

The government’s tendency to formulate national programs and strategies is positive, but lack of regional funding and support limit effectiveness. Schools and healthcare environments can play powerful roles supporting mental health, however, experience considerable challenges related to resourcing, and lack spaces and processes for multisectoral working. There is a pressing need to resource and support implementation and collaboration capabilities of the school community and healthcare sector to promote mental wellbeing and provide culturally engaged services.

## Introduction

Mental health requires attention for the well-being of people in general, and children and adolescents in particular. In this study, we explore child and adolescent mental well-being in Ethiopia in terms of the nature and extent of the problem, and policy support and recognition to prevent and address the issue. In Ethiopia, mental health problems are a considerable and growing concern, often underreported and underdiagnosed due to stigma and limited access to care. Depression, anxiety, and substance use disorders are prevalent, and mental health disorders are estimated to affect 12–25% of children (Ministry of Health, Ethiopia, 2016). Studies highlight the impact of factors such as migration, and gender norms (Girma et al., 2021), identifying particular vulnerability in populations affected by poverty, conflict, and displacement (Ministry of Health, Ethiopia, 2016; Girma et al., 2021; Mitiku et al., 2024; Hunduma et al., 2022; Hunduma et al., 2024; Demoze et al., 2018; Tirfeneh and Srahbzu, 2020; Tilahun et al., 2019; Ministry of Health, Ethiopia, 2021a,b,c). Specific challenges have been observed in school settings, with a quarter of adolescents reporting poor health-related quality of life (Hunduma et al., 2022; Hunduma et al., 2024). Additionally, research shows that adolescents who have experienced trauma, such as abuse or parental neglect, are at heightened risk (Mitiku et al., 2024; Demoze et al., 2018). There is a clear need for comprehensive mental health interventions, including in schools and communities, alongside ongoing policy development and strategic implementation. However, the country faces challenges in integrating mental health services into primary healthcare (PHC), despite efforts to expand mental health support and train healthcare providers in delivering culturally sensitive care (Alem et al., 2009; Hanlon et al., 2017). Challenges such as insufficient financing, weak governance, and limited mental health professionals hinder effective service delivery, particularly for children and adolescents. The lack of coordination between government structures and inadequate training for health workers further complicates policy implementation at community level (Hanlon et al., 2017).

Mental health problems, including among children and adolescents, are driven by a range of factors including poverty, migration, gender norms, social capital, which individually and collectively undermine agency over healthy behaviors, limit access to services, and reproduce inequalities (Hunduma et al., 2022; Jones et al., 2020; Owens and Hadley, 2023). As Burns argues, the prevailing market-oriented neoliberal political orientation can lead to increased income inequality and reduced social cohesion, with deleterious impacts on mental well-being, especially among vulnerable groups including children and adolescents (Burns, 2015). Therefore, understanding the relationships between politics, poverty, inequality, and mental health outcomes requires a robust, evidence-based ‘political economy’ of mental health. Responding to general ‘policy inaction on the structural drivers of health inequities’ (Friel et al., 2021), we explored child and adolescent mental well-being in Ethiopia using political economy analysis (PEA) to provide a broader, synthetic view of child and adolescent mental well-being. PEA is a framework used to understand how political and economic factors shape policies and outcomes. It focuses on the distribution of power, resources, and interests within a society, and how these dynamics influence decision-making. Analysing these factors can improve understandings of mental health issues among children and adolescents, stimulating a system-wide approach supporting identification of structural and institutional features, and networks of influence on agency and behavior, and supporting identification of routes for change. ‘Structures’ generally refers to the broader systems that shape how society functions, and ‘institutions’ are considered as specific organizations or groups within those systems. These can be formal, e.g., schools and hospitals, or informal, e.g., family networks or community support systems. ‘Agency’ refers to the capacity of individuals or groups to act independently, make choices, and exert influence over their own lives and circumstances. In the context of this study, these dimensions refer to the ability of children and adolescents to make decisions regarding their mental health and well-being, in the contexts of external constraints such as socio-economic factors, stigma, and limited access to services.

In Ethiopia, mental health services for children and adolescents are limited, and many of the barriers to improving services are tied to political and economic structures. PEA helps identify how power dynamics, budget allocations, and the influence of various stakeholders—policymakers, healthcare providers, and international organizations—shape mental health policies and services. The lens supports examination of systemic causes, such as inadequate funding, social stigma, and competing priorities, which hinder effective service delivery. While there is no unifying definition, PEA focusses on the distribution of political and economic resources influence, considering power, interests, ideologies and institutions (Reich, 2019). For example, and as outlined, above, despite policy commitments to mental health, there are significant gaps in implementation due to lack of resources and coordination between different levels of government. Using PEA, we can examine how these challenges are rooted in power imbalances between federal and regional authorities, and how the allocation of financial resources to mental health services often competes with other national priorities. The aims were to describe the political, economic, social and policy contexts in which child and adolescent mental well-being exists; and analyse power, interests, intentions, and alliances, and the influence and resources key stakeholders bring to bear on decision-making processes related to child and adolescent well-being.

## Materials and methods

### Study setting

There have been dramatic improvements in population health in Ethiopia over the past 25 years. These have been attributed to a comprehensive PHC approach; combining disease programmes with health systems strengthening, community empowerment and multisectoral action. There have been major investments in community-based PHC, expanding Community Health Workers (known as Health Extension Workers, HEWs) (Assefa et al., 2019; Negussie and Girma, 2017; Tadesse et al., 2022). HEWs promote health, including education, screening, prevention, and some clinical interventions. Since 2003, more than 42,000 HEWs have been trained and deployed in the country (Bowser et al., 2023; Caglia et al., 2014). The Universal Health Coverage (UHC) service coverage index has improved from 13 (very low coverage) in 2000 to 35 (low coverage) in 2021 (World Health Organization, World Bank, 2023). Child mortality has fallen significantly, from 141 to 46 deaths per 1,000 live births 2000–22 (IGME, UNICEF, World Health Organization, World Bank, United Nations, 2024), and life expectancy has increased from 51 to 68 years over the same period (World Health Organization, 2024). Despite improvements in population health, challenges persist, such as the need for better adaptations to manage noncommunicable diseases (NCDs) including mental health disorders and in coverage, access, sub-par quality of care, and high out-of-pocket expenditure (Assefa et al., 2020). Communicable diseases and maternal and child mortality and morbidity remain high, and NCDs including mental health disorders, and injuries are a serious problem (Li et al., 2022; Ministry of Health, Ethiopia, 2020a,b). These suggest that mental health problems for children and adolescents need specific attention within these broader improvements.

### Analytical framework

**Figure 1:**
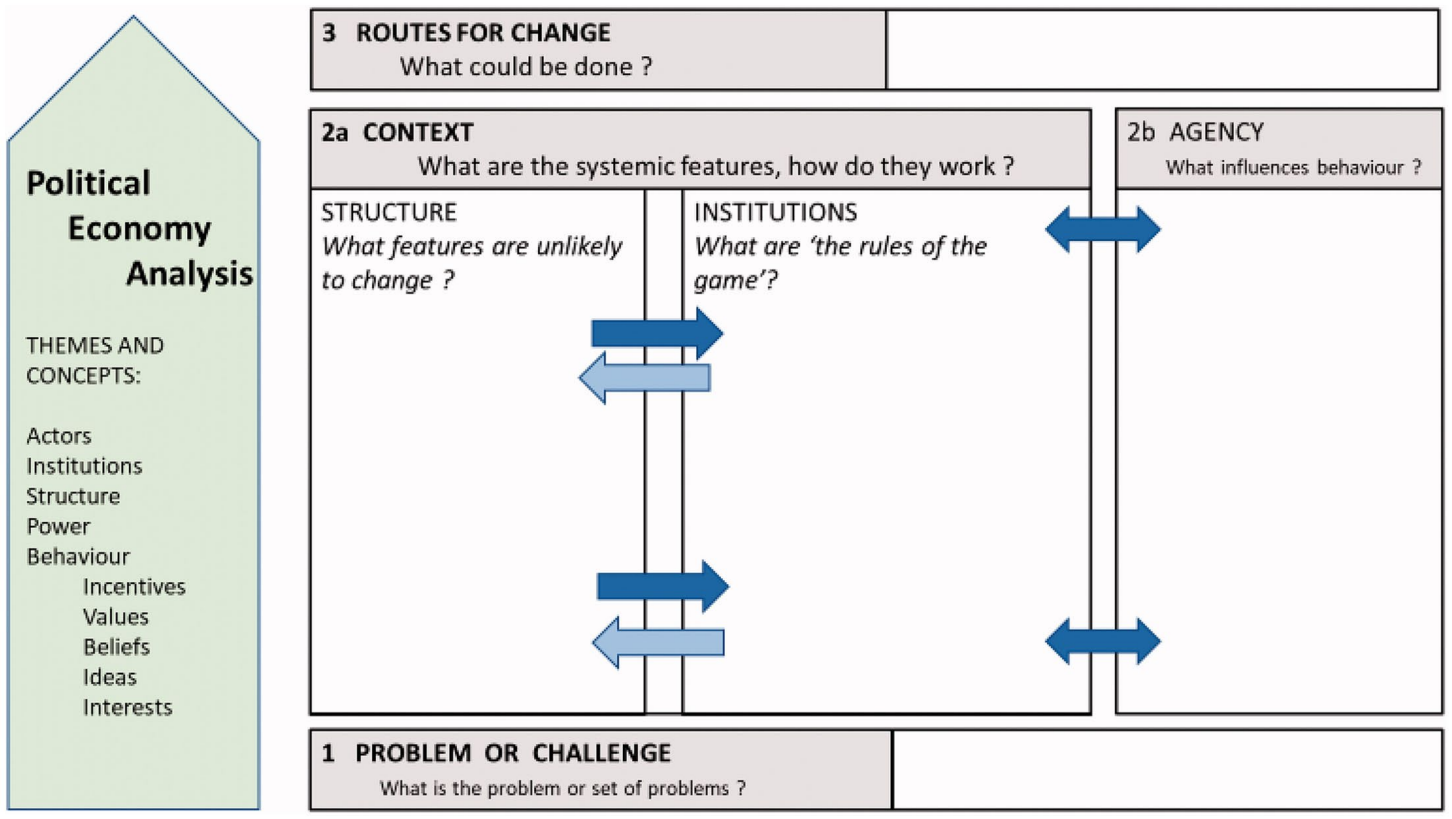
Political economy analysis framework (source: Andreas et al., 2022).

In this study, we use a PEA framework to understand how different factors influence child and adolescent mental well-being. As outlined in the previous section, PEA focuses on structures, institutions, and individual actions and their influences, dynamics, and interactions, in shaping outcomes. While there is no unifying definition of PEA or its key constructs, there is little ambiguity that both ‘structures’ and ‘institutions’ are non-agential phenomena (Burns, 2015; DFID, 2009; Fleetwood, 2008a,b; Harris, 2014). For the purposes of the study, we outlined the main analytical components of PEA. We considered ‘structures’ as the deeply rooted factors that influence society. These include, e.g., history, geography, economy, and political systems, which influence institutions, and which are difficult or impossible to change (Harris, 2014; Andreas et al., 2022; Heller et al., 2024). ‘Institutions’ were defined as the formal and informal organised systems, conventions, norms and values that are devised by humans, and that shape social interactions; in plain language, ‘the rules of the game’. These include the health system, schools, and the family, and are more flexible and amenable to change through policy or societal shifts (Fleetwood, 2008a,b). Aligned with Fleetwood’s conception, we considered structures and institutions as having causal powers influencing behaviors and outcomes (Fleetwood, 2008a,b). Finally, ‘agency’ refers to individuals’ ability to take action (health-related or otherwise) (Giddens, 1979). We examined how key stakeholders, including service users and providers, act based on their resources, power, and interests to influence mental health outcomes for children and adolescents. Moreover, a central theme in PEA is the notion of *reflexive deliberation* between structures, agents and institutions, whereby agency (individual actions) reflects and reinforces structure (social context) (Fleetwood, 2008a,b). We adopted a specific, problem-oriented approach framing these characteristics as holding the potential for positive change, which is politically feasible (Andreas et al., 2022) (Figure 1).

The design was also configured acknowledging calls to ensure awareness of the implicit influences on policy discourse (Andreas et al., 2022). We therefore sought to elicit and combine *explicit knowledge* on the subject, i.e., that which is formal, codified and written down, with *tacit knowledge,* which, while harder to codify, includes the experiences and perspectives of different actors’ multiple, situated meanings, roles, and realities of the subject. This approach was deemed appropriate given that mental health is subject to relatively higher detrimental and stigmatising information than other areas of health (Lee et al., 2015). According to this approach, we conducted a cross-sectional qualitative study comprised of: (a) a non-exhaustive review of the literature on child and adolescent mental well-being in Ethiopia, and (b) key informant interviews (KIIs) with government officials, representatives of nongovernmental organizations (NGOs), health professionals, technical experts and academics holding formal roles in child and adolescent mental well-being policy, strategy, and service delivery. *Tacit knowledge* was primarily gathered through the KIIs providing in-depth insights into lived experiences, personal perspectives, and subjective understandings of mental health issues. Participants shared personal knowledge, challenges, and the nuances of policy implementation that are often not codified in official documents. In addition, we reviewed the literature as a source of *explicit knowledge* that offered insights into the challenges and experiences related to child and adolescent mental health in Ethiopia. By combining both explicit (formal, written knowledge) and tacit (informal, experiential knowledge) sources, the study aimed to develop a comprehensive understanding of the factors affecting child and adolescent mental well-being in Ethiopia. This approach allowed us to consider both official policies and strategies, as well as the nuanced perspectives and experiences of those directly involved in service delivery and policy implementation.

### Data collection

A review of peer reviewed literature on health and education systems and policy for child and adolescent mental well-being was performed. We also accessed ‘grey’ literature from the ministries of health and education and other government sectors as relevant. Various policy and strategy documents including governmental and NGO documents were reviewed. The literature review was conducted systematically by searching several databases, including PubMed, Scopus, and Google Scholar. We used keywords such as ‘child and adolescent mental health in Ethiopia,’ ‘mental health policy,’ ‘mental health services,’ ‘primary healthcare and mental health,’ and ‘youth mental health interventions in Ethiopia, ‘socio-cultural status of Ethiopia’, ‘political and economic status of Ethiopia’, ‘urbanization in Ethiopia’. We broadly searched for relevant studies published in the last 10 years, focusing on child and adolescent mental health within the Ethiopian context. We reviewed both peer-reviewed articles and grey literature, including government reports and NGO publications to capture a comprehensive view.

For the KIIs, participants were purposively selected to represent a range of stakeholders involved in child and adolescent mental well-being in Ethiopia, including government officials, development partners, NGOs, academia, and civil society working in the education and health sectors in the Ethiopian context and with formal roles and interests in child and adolescent mental well-being. A total of 9 participants were interviewed, with seven male and two female representatives. The sample was designed to ensure the representation of various government organizations at both regional and federal levels, local and international NGOs, academia, and professional institutions. Letters were sent to the Minister/Head of Organization describing the research and requesting a nominated representative member to participate. Nominated individuals were then provided with a participant information sheet and consent form. Those expressing interest were provided with details for the team, and a convenient date, time, and venue were arranged for the interview. All interviews were conducted between June and November 2023 in person at the offices of informants, or by telephone where necessary. Interviews lasted 40–120 min.

The interviews sought to elicit insights into the national and local contexts in which children and adolescents live, which conditions their lives, and impacts their mental wellbeing. Interviews were semi-structured and performed using pre-prepared topic guides, which included questions on the health and wellbeing of children and adolescents, national policies and strategies for health and education, and the impacts of these policies. Interviews progressed until there was a reasonable degree of thematic saturation. Saturation was reached when no new significant themes were identified from subsequent interviews, indicating that the data collection had sufficiently covered the relevant areas of inquiry. This was assessed during the interviews by regularly comparing new interviews to previously collected data to ensure that no additional concepts or patterns were emerging. Interviews were audio-recorded and translated verbatim in the interview language Amharic, and thereafter into English for analysis. All transcripts were checked to ensure translation accuracy and quality.

### Data analysis

Selected documents were categorised, including their titles, authors, and year of publication for analysis. Each document was carefully read to familiarise with contents. Contents were categorised using deductive coding, based on a pre-set coding scheme aligned with the study objectives and analytical framework. Documents were analysed for what they explicitly stated (semantic) and for subtext to uncover implicit assumptions. To organise and analyse the interview data, and structure interpretation, we used framework analysis (Goldsmith, 2021; Ritchie and Spencer, 1994). Framework analysis is a systematic yet flexible approach that allowed for collaborative analysis within the research team (Gale et al., 2013). We followed the five main steps of framework analysis as follows. For Step 1: *Familiarization* involved an initial immersion of the researchers in the data by reading and re-reading the transcripts. This enabled us to develop an initial set of codes based on initial impressions. In Step 2: *Identifying a framework*, we then aligned the initial codes with the Andreas *et al* framework (Andreas et al., 2022), which provided a structured basis for further analysis. In Step 3: *Indexing*, we systematically coded all interview data, ensuring data were organised by the categories defined in the framework. In Step 4: *Charting*, the indexed data were organised into a matrix summarising ‘cases versus codes’ to identify relationships between themes and respondents. Finally, in step 5: *Mapping and Interpretation*, we analyzed variations and commonalities within the matrix to identify key patterns, commonalities, and abstractions linking themes and cases. Most of the steps progressed iteratively developing and applying the framework. Narrative data were managed in NVivo version 12. This software facilitated organization of large volumes of qualitative data and helped in systematic coding. In NVivo, codes were applied to segments of text that corresponded to specific themes and categories as defined in the framework. We used NVivo’s coding query tools to ensure consistency in applying codes across all data.

### Ethical considerations

The study protocol was reviewed and approved by ethical review boards at Addis Ababa University and the University of Aberdeen (Clearance references: AAUMF 03-008. 111/22/Psy and 20/06/2022 Committee for Research Ethics and Governance in Arts, Social Sciences and Business). Participant selection and recruitment involved several activities, including identifying eligible individuals, adequately explaining the study to potential participants, obtaining informed consent, and maintaining ethical standards with data collection, management, and analysis. Participant involvement was voluntary. For this reason, the team provided participants with adequate information and allowed sufficient time for individuals to make reasoned and free decisions regarding involvement. Participants were assured anonymity and that their identities would be protected. Potential participants were also informed that they were free to leave the study at any time and for any reason or no reason. All data were anonymised. All participants were given unique codes, and only the codes appeared on any stored data including transcripts, notes, and recordings. To further protect participant privacy and ensure data security, all linking data were stored separately on encrypted jump drives in a secure location accessible only to TH.

The study adhered to the parent programme’s Data Management Plan designed to protect the integrity and security of data, and the confidentiality of research participants. Accordingly, we took several specific measures: all data were securely backed up daily to ensure integrity; all data were stored only on password-protected computers; and were encrypted before being transferred electronically using secure file transfer; linking information, such as participant identifiers, were stored separately to the main dataset in a secure, restricted location, and access was limited to essential use; and that data. Following completion of data collection in the parent project, all linking data will be destroyed to further protect participant confidentiality. Finally, following the completion of the project, all data will be archived for a period of five years, and all non-sensitive project records will be made available in an open-access repository within six months under a Creative Commons license.

## Results

The results are presented according to the three analytical domains: Structure, Institutions, and Agency. Each domain represents a critical layer of influence on child and adolescent mental well-being. First, we examine the political, economic, and social structures shaping mental health policy and access to services. Second, we explore the role of institutions such as the family, education system, and health system in supporting and/or limiting mental health outcomes. Finally, we discuss the agency of key stakeholders, including children, parents, teachers, and health professionals, and the ways they navigate the constraints and opportunities within these structures and institutions. The thematic analysis is presented below, illustrated with verbatim quotes. A profile of the respondents is contained in Table 1, and the thematic and stakeholder analyses are summarised in Table 2.

**Table 1:** Profile of respondents (*n* = 9).

Sector	Number
Education	1
Addis Ababa City Administration Health Bureau	1
Psychiatrist	1
Ministry of Women and Social Affairs	1
University, child and adolescent mental health specialist	1
International NGO	1
MOH, Non-Communicable Diseases and Mental Health Desk	1
MOH, Reproductive and Family Planning Youth desk	1
NGO	1
Total	9

**Table 2:** Thematic and stakeholder analyses.

(A) Thematic analysis		
Construct	Situation/need	Response
Structure	Multidimensional social disadvantage: poverty, child and adolescent labour and early marriage, conflict, weakened social bonds; drugs and alcohol Stigma and discrimination exist on many fronts Violence normalised: widespread accounts across domestic and school environments	Major shifts in policy support and recognition Policy support in health and education Child rights important aspect of policy
Institutions	Education system faces many challenges: poor conditions for teachers; overcrowded classrooms; low access, high dropout, drugs Health system similarly challenged in terms of personnel and facilities to address the burden of child and adolescent mental wellbeing. Health system challenges in terms of low awareness, poor working conditions	Limited budget/inadequate staffing Limited leadership, governance and management Low intersectoral collaboration and coordination Accounts of services: lacking, inconsistent and duplication cf. development partners and multiple vertical programmes Strong M&E but accounts of manipulated and limited data and research
Agency	Structural disadvantage combined with low quality/access to health and education services dislocating children and adolescents from education system (primarily) and health system support for mental well-being	Serious and multiple implementation gaps constrain service users and providers from responding to the problem of child and adolescent mental well-being

(B) Stakeholder analysis				
Stakeholders	Power	Interests	Influence	Resources
Teachers and educators	Low	High	Low	Implementation capabilities Local actor power
Health workers	Low	High		
Children and adolescents	Low	High	Low	Grounded experience Local intelligence Local community entry
Parents and families	Low	High		
Local NGOS, civil society and faith-based groups	Medium	Low		
Researchers and technical agencies	Medium	Medium	Medium	Data/research capability
Regional planners and managers	Med/Low	Med/Low	Med/High	Technical capability Financial resources Political power
Federal health officials, policy makers and civil servants	High	Med/High		
INGO, donors and development partners	High	Med/High		

### Structure

#### Politics and political contexts

Ethiopia is a federal republic with a diverse multi-ethnolinguistic population. Federalism was introduced by the Ethiopian Federal People’s Revolutionary Democratic Front (EPRDF) when the party came to power in coalition in 1995. The EPRDF established 11 ethnically defined states and two city administrations organized into zones, districts and kebeles (the smallest administered units) (Federal Democratic Republic of Ethiopia, 1995). States have legislative, executive and judicial powers, and are responsible for public service delivery. Devolved administrations are financed through central block grants, regional revenues (land, business and income taxes), grants, loans, borrowing and development funding. While devolved administrations have significant powers, they are heavily dependent on central grant transfers and federal frameworks. Informants narrated operational contexts characterised by vastly inadequate budgets, and challenges with federal and regional level coordination, as severely undermining front-line services.

*They plan without resources…regarding psychosocial support…it says ‘to reach 1.5 million families’…it does not match the resources available…This budget itself has been reduced to non-existent (KII, Ministry of Women, and Social Affairs)*

Critical disconnects between policy goals and available resources was a recurring theme throughout the interviews. Several informants described how insufficient funding severely undermines implementation capabilities limiting service provision. Lack of financial resources results in staff shortages, inadequate training, and an inability to provide necessary materials or infrastructure for mental health, leading to severe gaps in services. Moreover, financial difficulties exacerbate existing inequalities. As described earlier, regions outside the capital city, Addis Ababa, face greater challenges in accessing adequate mental health support. The result is a significant disparity in mental health service availability, with rural and conflict-affected areas suffering the most. This reveals lack of sustainable funding for key services as a key structural barrier hindering implementation of mental health policies. It also underscores the importance of aligning policy commitments with realistic budgetary allocations. Without sufficient funding, policies designed to support large segments of the population remain inaccessible and ineffective.

Ethiopia is considered politically authoritarian. In 2005, the country held what was lauded as the most democratic election in history. However, the dominant party met demands for self-rule with suppression and violence, imposing restricted rights and freedoms (Brown and Fisher, 2020). In 2018, following years of political dissent, protests, and crackdowns by the ruling party, Abiy Ahmed was elected as the fourth prime minister in EPRDF’s reign. Abiy introduced promising political and economic reforms, but developments have been reversed by COVID-19, ethnic tensions, and conflict (Kabeta, 2021). In 2020, a bloody conflict erupted between Tigray and federal allied forces with severe humanitarian, social and economic impacts: destruction of livelihoods, displacement, and a collapsed healthcare system in the North (Gesesew et al., 2021). A prominent theme in the interviews was the high prevalence of psychological distress among students in conflict-affected regions. This was corroborated in the literature, including with reports that up to two-thirds of students (59%) in conflict-affected areas report experiencing mental distress (Madoro et al., 2021). This underscores the profound psychological toll of conflict and displacement, with children and adolescents facing considerable emotional and mental health challenges.

*In crises-affected regions including Tigray and Amhara, we expect many children to suffer from mental health problems due to war and internal displacement (KII, civil society)*

Key informants, particularly from the education and civil society sectors, described the multifaceted impact of this distress on young people. Many children in these areas are exposed to trauma from witnessing violence, experiencing displacement, or losing family members. The consequences are far-reaching, impacting academic performance, social relationships, and long-term mental well-being. For example, one education expert noted that children living in internally displaced persons’ camps exhibit signs of chronic stress, including difficulty concentrating, increased absenteeism, and behavioral problems in school settings. Furthermore, mental health services are scarce in these areas, and many children are unable to access appropriate support due to stigma and logistical barriers. The lack of adequate mental health care is exacerbated by the ongoing conflict, where resources are focused on immediate humanitarian needs rather than long-term health interventions.

#### The economy

Ethiopia is a mixed market economy: most of the population is engaged in subsistence/semi-subsistence agriculture, together with services, and the economy has transitioned from state to private sector with aid conditionality for trade liberalization (Planning and Development Commission, 2020). Growth has averaged 10% per year over a decade, and the government plans for the country to reach lower-middle-income status by 2025 (Planning and Development Commission, 2020). Despite economic transition, Ethiopia remains one of the poorest and least developed countries in the world. COVID-19 slowed growth, caused high inflation, high unemployment, and put significant pressure on the economy. The civil war has also had a heavy toll on economic development (Gebreyesus et al., 2023). Inflation was 31% in 2023 (UNICEF Ethiopia, 2023a,b). About 69% of the population is multi-dimensionally poor and there is widespread poverty in rural areas (UNDP, 2023). Urban poverty is driven by migration, overcrowding, social fragmentation, crime, violence, and youth unemployment (Mekasha and Tarp, 2021). In 2020, urban unemployment for youth (15–29 years) was 25.7% (18.9 for men and 31.7% for women) (Central Statistical Agency, 2020). Poverty was frequently cited by informants as significantly determinantal to child and adolescent mental health. One education sector expert highlighted how overcrowded homes, where basic needs like space and stability are scarce, contribute to constant stress, which can manifest in anxiety, depression, and other mental health issues, in turn influencing children’s ability to focus on education, develop emotionally, maintain well-being and envision a positive future. Poverty exacerbates exposure to adverse experiences like family conflict and limited access to healthcare. Furthermore, stigma and economic barriers prevent many families from seeking professional support, despite need. These findings point to the need for integrated interventions, recognising that poverty is not only an economic issue but also a critical determinant of mental health in Ethiopia.

*Poverty itself has its own contribution to mental health problems…Children who live in a house with insufficient space cannot see the future (KII, education sector expert)*

Ethiopia’s reliance on foreign aid, with development partners heavily involved in health, education, and governance, presents both opportunities and challenges. While development assistance aims to support growth, reduce poverty, and stabilise the region, long-term dependence on foreign aid may *hinder progress* toward self-sufficiency and sustainable economic development, impose certain strategies and priorities, and ineffectively influence foreign and domestic policies (Lie, 2019; Sande Lie, 2024). Despite being a significant recipient of development assistance since the early 1950s, the Human Development Index (HDI) for Ethiopia is low at 0.5 in 2022, and ranked 176th out of 196 countries and territories (UNDP, 2024). As one informant from an INGO described, many aid-driven mental health programs in Ethiopia are standardised with limited contextualization necessary for sustainability. Moreover, short-term programmes reliant on donor funding struggle to be sustained or adapted to the unique needs of the population, particularly in the mental health sector. The national mental health strategy aligns with the agendas of, and is developed in consultation with, development partners (Ministry of Health, Ethiopia, 2021a,b,c). The national plan aligns with the WHO Global Comprehensive Mental Health Action Plan 2013–20 (World Health Organization, 2013), the Sustainable Development Goals (SDGs) (United Nations, 2024), the UN Convention on the Rights of People with Disabilities (United Nations, 2006), and the UN Convention on the Rights of the Child (United Nations, 1989). However, key informants highlighted weak coordination between development partners and the government, which often leads to fragmented services. The lack of integration between donor-supported programs and national systems hampers the long-term effectiveness of mental health initiatives, particularly in addressing the specific needs of children and adolescents.

*Most organizations, let us say [major INGO], have these different programs on mental health and psychosocial support…it’s pretty much the same package that they have used in several other contexts […] it’s not very coordinated, and they cannot necessarily sustain it if they have short-term funding (KII, INGO)*

#### Social and cultural contexts

Ethiopia’s rich cultural diversity shapes the way mental health is understood and treated. Ethiopia is an ancient civilization and the birthplace of humankind, with a multitude of ethnicities, religions, and cultures. Many cultural, spiritual, and supernatural beliefs regarding the symptoms and causes of mental illness were reported in the literature; including magic, curses, possessions, and sin (Abera et al., 2015; Asfaw, 2015; Jacobsson and Merdasa, 1991; Madu and Ohaeri, 1989; Mekonen et al., 2022; Molvaer, 1995; Monteiro, 2013; Monteiro and Balogun, 2014). Widespread stigma and discrimination related to mental health problems were also surfaced in the review, with religious practices reported as preferred treatments, and health service support seen as a last resort (Mekonen et al., 2022; Girma et al., 2022). Informants corroborated and expanded on this, narrating fear and stigma as powerfully discouraging care seeking, particularly for children and adolescents, and contributing to widespread lack of awareness and/or traditional beliefs among service users and families. Informants highlighted how traditional beliefs create significant barriers, with families often avoiding medical treatment in favour of religious or spiritual interventions. Respondents also described how the situation is exacerbated by lack of available and accessible services. The proportion of Ethiopians with health insurance is relatively low, for example, at around 28% (Merga et al., 2022), underscoring access to care as a significant challenge, especially for vulnerable groups. This highlights multiple, converging barriers to access of mental health services for children and adolescents.

*People with mental health problems are not taken seriously…they are associated with religion and considered a curse. The service is not available…and stigma and fear create a compounding problem (KII, University, authors’ emphasis)*

*…if children face a physical health problem, they go to a health facility, but if it’s a mental problem, they do not go (KII education sector expert)*

Harmful gender norms, including female genital cutting, circumcision or mutilation (FGC/M) and early marriage, have severe implications for the health and well-being of girls and women in Ethiopia. National data indicate that 65% of women are circumcised; nearly half (49%) undergoing the procedure before the age of five (Central Statistical Agency, Ethiopia, ICF, 2016). FGC/M has disastrous effects on girls’ health and well-being (Chuta et al., 2019). While prevalence is in decline (2000–16), reductions are modest (Alemu, 2021) and uneven: the highest prevalence being in Somali (99%) and lowest in Tigray (23%) (Central Statistical Agency, Ethiopia, ICF, 2016). Early marriage is a further ancient practice. Studies report 49% of women married before age 15, and 83% before 18 years, with extreme psychological and emotional impacts (Asrese, 2014). Studies have reported compound impacts and trauma associated with very early marriage negatively impacting virtually all aspects of psychological well-being—depression, anxiety, positive well-being, vitality, and overall health (John et al., 2019). Driven by poverty as well as tradition, early marriage itself drives migration as girls flee to urban areas to escape it (Tekile et al., 2020). The government has committed to ending child marriage and FGC/M by 2025, through the National Alliance to End Child Marriage and FGM/C (Marshall et al., 2016; Ministry of Women, Children and Youth, 2019), which was described as active and visible at grassroots levels. However, despite efforts, deep-rooted cultural and social factors continue to perpetuate these harmful practices. As one informant from the Ministry of Women and Social Affairs stated, there is significant advocacy and awareness-raising in local communities about the negative impacts of early marriage and FGC/M, which are central to the government’s commitment to ending these practices.

*We have pledged to drastically reduce and eliminate child marriage at the national level by 2025…There is a lot of advocacy work, especially on what the community thinks about early marriages and FGM (KII, Ministry of Women, and Social Affairs)*

#### The environment: demography, and geography

**Figure 2:**
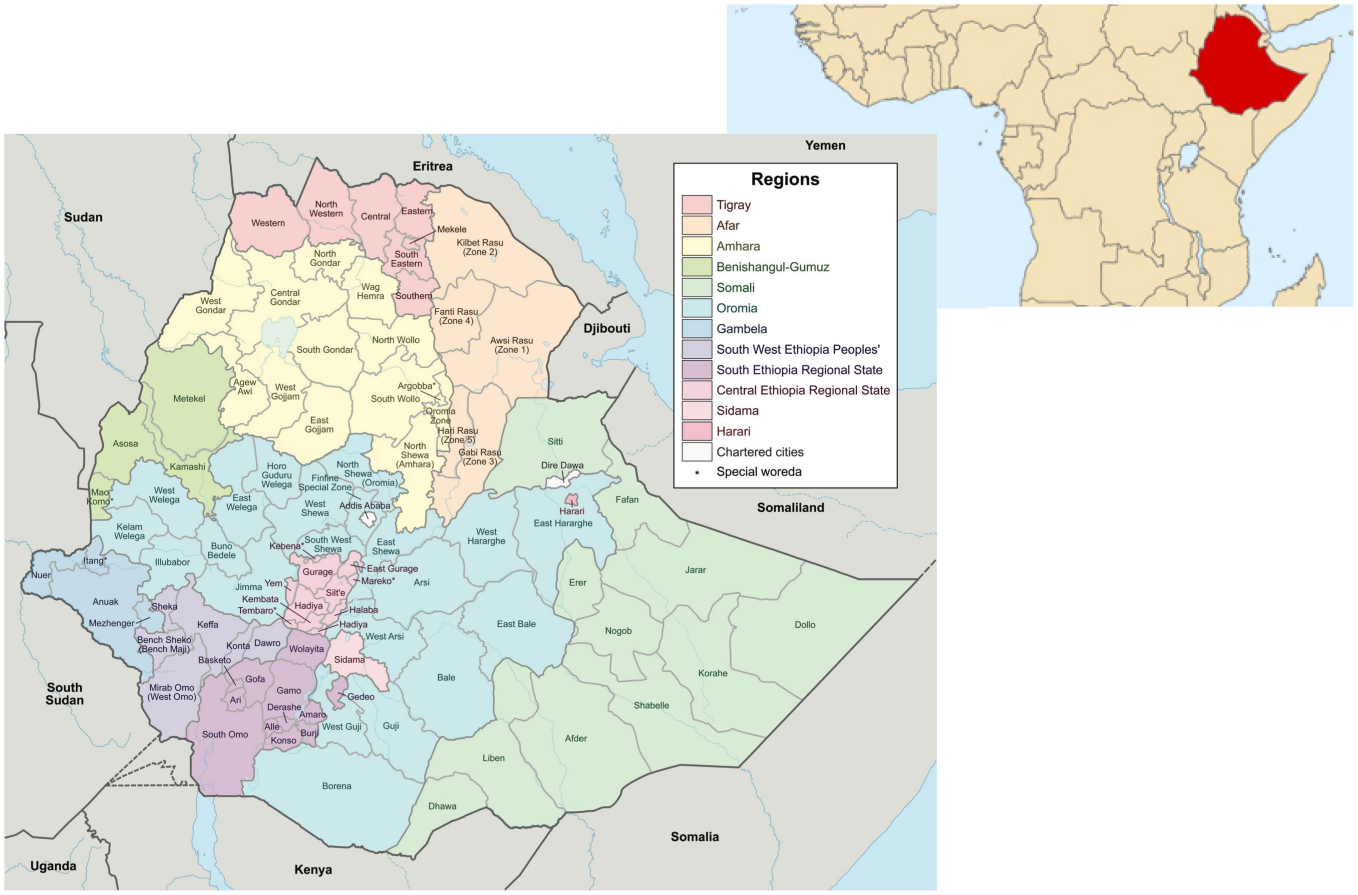
Map of Ethiopia, including location map (source: wiki commons https://commons.wikimedia.org/wiki/Maps_of_Ethiopia).

Environmental challenges in Ethiopia, such as urbanization, climate change, and demographic factors directly impact child and adolescent mental wellbeing. Ethiopia is one of the largest countries on the continent, covering 1.11 million km2 (Figure 2). With a population of 126.5 million, Ethiopia is the second most populous country in Africa (World Bank, 2024). The population is predominately young: children under 15 years comprise 44% of the population, the median age is 19, and there is a high total fertility rate of 4.6 (Central Statistical Agency, Ethiopia, ICF, 2016; Ethiopian Public Health Institute and ICF, 2021). Despite this demographic, Ethiopia remains one of the least urbanized countries in Africa, with only 23% living in urban areas, although it is also the fastest urbanising country on the continent (World Bank, 2024). With over 4 million dwellers in 2017, the capital city Addis Ababa is home to about a quarter of the urban population. The city faces a significant housing shortage, despite housing development (Larsen et al., 2019), which is compounded by overcrowding, noise pollution, and poor sanitation; factors that negatively impact children’s mental well-being. Respondents described many physical and environmental hazards for children in urban centres: overcrowding, noise pollution, and poor sanitation: *Children who live in a house with insufficient space cannot see the future (KII, education sector expert)*. This was supported by studies showing urban children as twice as likely to suffer mental health problems (Mitiku et al., 2024). In addition to urban challenges, Ethiopia is highly vulnerable to climate-related issues, experiencing extreme weather events such as severe droughts, at least twice per annum, together with regular, severe floods (Bezu, 2020). While the country has minimal emissions, it has taken bold climate action. The Climate-Resilient Green Economy Strategy (CRGE) in place since 2011 aims to drive low carbon development (Federal Democratic Republic of Ethiopia, 2011). Climate themes were not recounted by respondents, and relatively little literature was sourced. Nevertheless, some sources cited limited climate action policy and strategy (Bhopal et al., 2021; Fazzini et al., 2015), rising mortality and morbidity due to floods and extreme heat, vector- and water-borne diseases, meningitis, and pollution-related respiratory disorders (Simane et al., 2016), vulnerabilities across; health, water and agriculture, and interconnected environment and emotional wellbeing including in young adults (Simane et al., 2016; Freund, 2023; UNICEF Ethiopia, 2023a,b; Cooper et al., 2019).

### Institutions

#### The family

The family’s role was reported overall as critical in shaping how mental health is perceived and addressed. Family practices and norms regarding children in Ethiopia are deeply rooted in traditional values, with a strong emphasis on respect, obedience, and contributing to household duties, including farming and domestic work (Abebe, 2008; Kassa and Abajobir, 2018). Gendered family values and norms are instilled at a young age, and reinforced during adolescence and adulthood, often through verbal and physical punishment (Chuta et al., 2019; Poluha, 2004; Pankhurst et al., 2016). Violence in families was widely reported (Chuta et al., 2019; Pankhurst et al., 2016; African Child Policy Forum (ACPF), Save the Children Sweden, 2006a,b; Save the Children Sweden, 2005). National data indicate 99% of children experience physical punishment, psychological humiliation, and sexual violence in the home, and in schools or the community (African Child Policy Forum (ACPF), Save the Children Sweden, 2006a,b). For many families, these practices were reported to be justified as appropriate forms of discipline, and further exacerbated by poverty and limited access to supportive services (Desta et al., 2017). Limited referral, case management, legal and justice services were also reported (Jones et al., 2021). Key informants recounted the devastating, life-long consequences of widespread improper childrearing, early maltreatment, and abuse, including how these were amplified during COVID-19, and noting that children who experience early maltreatment, including abuse and neglect, are more likely to develop mental health problems, such as depression, anxiety, and developmental disabilities. One psychiatrist pointed out that many patients they encounter, particularly those with autism and mental retardation, had experienced inappropriate discipline and abuse during childhood. Key informants also recounted that these experiences are compounded by poverty, which often forces children into domestic labour, carrying burdens far beyond their emotional and physical capacity. Research also reports the impacts of child labour with all dimensions of psychosocial wellbeing, stress, self-esteem, and supervision, significantly associated with children’s working conditions (Fanton d’Andon et al., 2022).

*Most patients I encountered have developmental disabilities such as autism and mental retardation as they faced inappropriate discipline and abuse during childhood (KII, psychiatrist)*

*There are children who got stressed at a young age due to carrying a burden or workload beyond their shoulders (KII, psychiatrist)*

Informants also highlighted that, while awareness is growing, mental health problems are still denied or ignored by many families, owing to overall low mental health literacy and cultural reluctance around psychological conditions. Denial is often rooted in the stigma surrounding mental health, where symptoms are dismissed as either a spiritual issue or a moral failing, and where seeking professional help is seen as unnecessary or shameful. Denial notwithstanding, evidence of mental health harms experienced by primary caregivers of children and adolescents with mental health problems were also seen in the literature (Minichil et al., 2019). These issues perpetuate a cycle of misunderstanding and maltreatment with lasting consequences on child and adolescent development, well-being, and with consequences in families and communities, which in turn further impact children and adolescents, in non-linear, reciprocal mechanisms of influence. Despite the efforts of various institutions and policy changes, including advocacy against child abuse and gender-based violence, familial attitudes towards mental health exert powerful influences. These data suggest more concerted efforts are needed to educate families about mental health and promote supportive, non-violent child-rearing practices.

*Even though there is a greater understanding and awareness [of mental health], most parents tend not to accept the problem or deny the reality (KII, Psychiatrist)*

### Education system

Ethiopia has made substantial progress expanding access to education in recent years. The Education Policy of 1994, and the recently revised Education Development Roadmap focus on access, equity, equality, and quality of education (Ministry of Education, 2018; Ministry of Education, 1994), with budgetary increases of 60% from 2016/17 to 2020/21 (UNICEF Ethiopia, 2021). The number of primary schools has tripled (to nearly 35,000 in 2017), primary enrolment tripled between 2000 and 2016 (UNICEF Ethiopia, 2018), and net primary and middle grades enrolment (grades 1–8) was 89% in 2021/22 (Ministry of Education, 2022; Young Lives, 2012). Despite progress, challenges in education quality and infrastructure persist, and outcomes are poor. Learning poverty, the proportion of children not able to read and understand an age-appropriate text by age 10, is estimated to be 90% (World Bank, 2019). High dropout rates are another major concern, with only about half of young people completing grade 5 (primary schooling lasts 6 years), and upper-secondary school enrolment is under 10% (Jones et al., 2019). Dropouts are driven by poor infrastructure and pedagogical quality, ineffective leadership, overcrowded classrooms, poorly trained teachers, and low salaries particularly in rural areas were also reported in the literature (Aurino et al., 2014; Cueto et al., 2020; Frost and Rolleston, 2013; Shishigu, 2015). The pupil-to-classroom ratio was 54 in 2021 (grades 1–8) (Ministry of Education, 2022). One informant from Somali region described as many as 70 students sharing a single teacher, severely limiting teaching and learning.

*[In] Somali region, you saw 70 students with one teacher in the classroom (KII, INGO)*

Educational challenges have direct implications for the mental health of children and adolescents. Informants reported that teachers’ ability to provide psychosocial support and health education to students is hindered by insufficient human and financial resources including low motivation, poor salary, and overcrowded classrooms. Many teachers are overburdened, with large class sizes and limited support, increasing stress levels among both teachers and students. Several informants also noted widespread absenteeism exacerbated by the conflict. The situation is exacerbated by widespread corporal punishment in schools reported in the literature (Jones et al., 2019) with three-in-four students having witnessed it (Ogando Portela and Pells, 2015), and with deeply deleterious impacts on mental health, educational performance, attitudes towards school, self-esteem, cognitive and socio-emotional development (Jones et al., 2019; Ogando Portela and Pells, 2015; World Health Organization, 2021). Key informants confirmed violence as widespread in schools, with low awareness among teachers, especially for special needs students, highlighting prevalent abuse and the psychological toll it takes on students.

*If children go to school, they will be victims of violence (KII, University)*

The impact of gendered bullying, substance abuse, and violence in and around school environments further compounds risks to mental well-being. Health sector respondents narrated that schools, particularly in urban areas, are surrounded by khat (a flowering shrub with stimulant-like effects, widely consumed in the country) rooms and substance shops, which increase exposure to drugs and alcohol among adolescents. Despite regulations, informants recounted that the proximity of these establishments to schools exposes students to increased risk of substance misuse and mental distress. This environment not only reinforces unhealthy coping mechanisms but also deepens mental health vulnerabilities for those already at risk.

*There may be khat rooms, pubs, and shisha houses, especially around the schools, even though they have their own standards for how far they should be away from schools (KII health bureau)*

Furthermore, informants described the early phases of the COVID-19 pandemic, particularly lockdowns and other containment measures, as a ‘*psychological crisis’* for learners, with disruptions to education leading to social isolation, stress, and anxiety. Ongoing disruptions, coupled with economic instability, highlight the urgent need for systemic reform in Ethiopia’s education sector to address both the structural challenges in schools and the mental health needs of students. Specifically including around gender equality in schools, with trauma-informed approaches acknowledging widespread child abuse and exploitation, and stronger legislative and judiciary powers for prosecution (Mitiku et al., 2024).

#### Health system

There is broad policy recognition and support for mental health in Ethiopia. The first national strategy 2012/13–15/16 aimed to improve mental health service coverage through integrated PHC (Ministry of Health, Ethiopia, 2012). Ethiopia was one of the pilot sites to implement WHO Mental Health GAP action programme (mhGAP) (Ayano and Assefa, 2016). Following a five-year hiatus, the second national strategy (2020–25) was launched in 2021 again aligned with UN and WHO policy and strategy for mental health, development and child rights (Ministry of Health, Ethiopia, 2021a,b,c). This model promotes self-care as a central principle and activity, supported by formal systems and informal networks in an individual’s environment (Meshesha and Johnson, 2021). The HEP Optimization Roadmap was published in 2020 (Ministry of Health, Ethiopia, 2020a,b) aligned to the current Health Sector Transformation Plan (HSTP II), which cites mental health problems as a growing concern (Ministry of Health, Ethiopia, 2021a,b,c). The national adolescent and youth health strategy (2021–25) also includes mental health as a priority area (Ministry of Health, Ethiopia, 2021a,b,c). Central Ministry leadership and governance for mental health adopts an overall focus on improving access through PHC. The Federal Ministry allocates budgets and monitors and evaluates regional bureaus. Launched in 2003, the Health Extension Programme (HEP) is the government’s flagship PHC programme focussed on primary prevention through community-based services. Aligned with the national mental health strategy, in 2016, the Second-Generation HEP addressed NCDs included mental health disorders, and the Urban Health Extension Program (UHEP) has also been launched. The strategy focuses on school-based programmes. Positive impacts of HEP on adolescent mental well-being have been reported on child marriage, adolescent pregnancy, and education (Rudgard et al., 2022). Informants reiterated policy commitments, including improved metrics and monitoring.

*We have an indicator that includes psychosis, depression, bipolar disorder, epilepsy, and substance abuse. Another is…availability of services to children under the age of 18…this is a breakthrough (KII, Ministry of Health)*

Limiting these gains, significant *implementation challenges* were reported including insufficient financing, weak governance and leadership, and limited stakeholder involvement (Hanlon et al., 2017; Mekonen et al., 2022; Ayano and Assefa, 2016). Tensions between prescribed policy commitments and implementation realities and capabilities was a repeated and persistent theme in the interviews (and illustrated the complementarity of knowledge, located in subjective experience, with knowledge seen in documentary formats). Narrated in various terms: budget shortfalls, resource shortages, and lack of policy-implementation knowledge transfer at community and primary school levels, insufficient financing was seen as a critical issue and one leading to poor governance across levels. One Ministry official noted:

*We have a dearth of professionals…we could not afford to hire (KII, Ministry of Health)*

Inadequate personnel and infrastructure were widely documented. There is only one psychiatrist per two million population, 0.7 mental health professionals per 100,000 population (Ministry of Health, Ethiopia, 2012; World Health Organization, 2020; Borges et al., 2010), and only one dedicated mental health hospital with outpatient units available in only two hospitals, all of which are located in capital city (Yitbarek et al., 2021) leaving rural and regional areas facing significant shortages and barriers to access. Informants noted for example how expanding services via HEWs has been unsatisfactory with inadequate training leaving HEWs without core capabilities to screen and make referrals. The HSTP II itself acknowledges a *virtual absence* of child and adolescent mental health services (Ministry of Health, Ethiopia, 2021a,b,c). Despite school-based mental health strategies, informants expressed frustration with the lack of implementation at local levels, with teachers and health workers often unaware of the mental health components of the policy. Poor coordination between federal and regional political structures were described in the challenges *of* scaling specialized care within general health service frameworks.

*…the strategy [Ethiopian School Health and Nutrition] is not seen when it goes down and works with primary schools (KII, psychiatrist)*

*Our ability to influence regional administration has been limited. So, when you go down to regional offices, each region is autonomous. They prioritize on tasks based on their assessment. Therefore, mental health is still not given a priority (KII, Ministry of Health)*

By improving coordination between federal and regional authorities, increasing budget allocations for mental health, and prioritizing training and infrastructure development, Ethiopia could significantly improve its ability to meet the mental health needs of its children and adolescents. Addressing these challenges require strong leadership, better resource allocation, and a shift in policy priorities to ensure mental health services are not sidelined in the broader public health agenda.

### “Agency”: stakeholder power, interests, and resources

Informants described multiple constraints to child and adolescent agency over their mental well-being, pointing to poverty as a key determinant. Children, especially those from low-income background, are often forced out of school and into income generation. Working environments impose environmental hazards, heighten stress, and impair educational performance, further perpetuating the cycle of disadvantage (Jones et al., 2019; Frost and Rolleston, 2013). Many children are also tasked with caring for relatives, which exacerbates the burden on their time and emotional capacity, leaving little space for healthy mental development. As one education sector expert noted, substance use, such as benzene inhalation among adolescents, is rampant. This can be seen as a coping mechanism that reflects the trauma and environmental stressors faced daily. An entrenched drug crisis can be considered as existing in a vicious cycle, where substance abuse only deepens the mental health crisis, reflecting and reinforcing trauma among vulnerable children and adolescents from poor families and households.

*We see children and teenagers using benzene everywhere (KII, education sector expert)*

While teachers and health professionals are positioned to play critical roles in supporting students’ mental well-being, structural constraints undermine their effectiveness. Informants shared how teachers’ working conditions are particularly challenging, with low pay, high stress, and overcrowded classrooms. These factors limit their capacity to provide meaningful mental health support, despite policy frameworks that promote mental health in schools. A key informant from the Ministry of Education pointed out that teachers often regret joining the profession due to poor working conditions, and that this stress detracts from their ability to be effective educators. The lack of adequate training and understanding of mental health issues further impedes teachers’ ability to address mental health needs in the classroom, even though they are often the first point of contact for students in need of support.

*I see teachers often regret joining profession as there is little benefit attach to it. I personally know a teacher who is unable to support his family and was under stress. As a result, he could not do his job effectively (KII, Ministry of Education)*

Informants provided granular accounts of *compounding* vulnerabilities. Informants described how widespread stigma and lack of knowledge about mental health issues within families, schools, and communities severely limit children and adolescents’ agency to seek help. Stigma surrounding mental health problems, coupled with a lack of resources and support services, means that many children suffer in silence, unable to access care. On the supply side, while the Ministry of Health is a major actor in mental health policy and strategy, it has limited capacity to *implement policy*, owing to lack of human resources and devolved governance. Limited human resources and inadequate governance at regional and local levels hinder mental health service delivery, leaving many communities underserved. As one informant from an NGO described, even though there are national strategies in place, without a clear, costed action plan at operation levels, strategies fail to translate into real-world contexts. These findings reveal how structural conditions (e.g., fragile economy) are reflected in institutional conditions (such as lack of teachers) and play out in services that are not capable of realising policy commitments and connecting with children, here around mental well-being in schools and society at large. It follows that collective remedial efforts can address these issues.

Regional and local autonomy was a clear feature of the accounts of the implementation of mental health policies. Regional authorities were criticized for prioritizing local needs over national strategies, leading to fragmented and disjointed service delivery. And operational actors pointed about the sheer lack of resources to render public services realistically with any chance of quality. Key power imbalances were evident whereby, on the one hand, national policies set the formal signals of priority and policy intent, however, on the other, their enforcement and prioritization at the regional and local levels was reportedly inconsistent, undermining mental health care access for children and adolescents.

*…what you need is a national policy, then you need to have these regional strategies … It’ll be developed, but there really will not be action planning beyond that. So, you do not have a costed strategy, and if you do not have that, you cannot really implement it (KII, INGO)*

*…more coordination between donors and the government, having a clear policy, and having clear regional strategies can help, because right now it’s very piecemeal. Everybody’s implementing their own package (KII, INGO)*

### Stakeholder analysis and routes for change

There was consensus among informants that any attempt to improve children and adolescents’ ability to manage mental health problems must focus on risk factors in home and school environments. To achieve this, informants highlighted the pressing need to strengthen intersectoral collaboration, particularly between the health and education sectors, as well as with media, schools, aid organizations, and other stakeholders. Supporting documents also reflected that line Ministries (health, education, finance, labour and social affairs etc.) can be powerful partners through intersectoral collaboration and ability to consider mental health across sectoral strategies and programmes (Ministry of Health, Ethiopia, 2016). While some spaces and processes were described as functional, several informants highlighted insufficient resources and fragmented processes as barriers to meaningful cross-sectoral working, with a general scepticism about capabilities to work across sectors. One key informant from the Ministry of Women and Social Affairs highlighted the structural barriers to integrating mental health into broader policy frameworks and the lack of concrete action in aligning efforts across sectors.

*Child and adolescent mental health has not been explicitly stated in our strategic plan…we have limited budget to work on children and adolescent mental health. Additionally, we found it difficult to mainstream such problems into lower-level administration as there is no structure to accommodate such programs…the sector has a very low tendency to work together…that interoperability is very low (KII, Ministry of Women and Social Affairs)*

To improve service responses and better integrate mental health initiatives, implementation strategies need to be developed and coordinated across multiple sectors. Key informants emphasized the importance of increased collaboration between donors and the government to ensure that mental health programs not only align with national strategies but are also adapted to local contexts. Strengthening these relationships and establishing shared goals can ensure that mental health initiatives are sustained, contextualized, and relevant to the communities they seek to serve. Key informants also highlighted mobilizing and empowering communities. Structures like child rights committees and the National Child Parliament was identified as a key mechanism for addressing mental health at the grassroots level. These organizations are able to address social issues, such as early marriage and FGC/M, and can be powerful allies in the promotion of mental health awareness and early intervention. Informants also recommended improving school health programs and underlined the importance of strong leadership to implement mental health programs in schools. Key informants asserted that such initiatives should be backed by leaders and with the inclusion of health professionals in school settings. Additionally, they argued that professional training should be given to health workers, parents, and teachers. Both the literature review and interviews indicated several spaces to negotiate and expand claims for child and adolescent mental health. Notably among these were Child Rights Committees and the National Child Parliament, described as widely operational and functional. At the same time, informants held the view that expanding recreational places, improving school environments, and enhancing the socio-economic status of households is crucial.

*…they [child rights committees] are functioning properly at the zone and district levels. For example, if an early marriage takes place in a place, the matter will be brought directly to the representatives of the children’s parliament, and then it will be brought to the zonal office of women and children…they have a unique role in solving existing social problems (KII, Ministry of Women and Social Affairs)*

Moreover, informants stressed the need for evidence-based practices and improved data collection to guide mental health policies. While some informants held the view that this would help improve existing programs, others were more skeptical with politicization of data candidly described. Establishing reliable, consistent data systems will allow for better tracking of outcomes, inform policy development, and ultimately ensure that mental health initiatives are effective and responsive to the needs of children and adolescents. However, data manipulation and lack of transparency in reporting continue to undermine these efforts. Overcoming this requires the establishment of stronger data collection protocols and transparent reporting practices to ensure accurate data for policymaking.

*It would be good to measure…changes brought about by the implementation of national strategies and programs at societal and institutional levels (KII, health science college)*

*If you go to the head of a children’s department and tell him how many children there are in the country, he will not know. The number of children at risk is similarly unknown. Data is manipulated. Not only this, but you will also be denied access to information (KII, Ministry of Women, and Social Affairs)*

*…what we see in the official figures may or may not be reality…Shall I inflate the numbers and make other people think that I am doing well so that they can support me more? Or do I face the reality and expose it so that others can come and help us improve the poor quality? These are the two basic determinant questions (KII, NGO)*

By including informants from diverse backgrounds, we aimed to provide a comprehensive view of the current mental health landscape. The differing perspectives add depth to the analysis and underscore the importance of considering local contexts when developing and implementing mental health policies. To ensure representativeness, a diverse sample of informants was selected, and whose narratives were analyzed and arranged with reference to the document review to situate subjective perspectives within a wider context. Participants included government officials, NGO representatives, health professionals, educators, and civil society members from various regions. A range of perspectives offered unique insights into the challenges and opportunities in their respective locales. For example, health professionals emphasized the lack of infrastructure and trained staff, whereas education sector experts focused more on the psychosocial impacts of the school environment and the challenges faced by teachers in addressing mental health issues. Significant differences were seen in perspectives of central versus regional stakeholders. Central level officials emphasized alignment of strategies with international frameworks, pointing to commitment and policy vision at national level. However, regional informants noted substantial disconnects between policy intentions and on-the-ground realities. While central government has made significant strides in developing mental health policies, regional authorities often cited insufficient funding, lack of trained personnel, and autonomy in resource allocation. This underscores lack of coordination, financial resources, and regional capacity result in fragmented service delivery and uneven access to services. The effectiveness of national policies can be improved by addressing these disparities and strengthening coordination between central and regional authorities.

## Discussion

**Figure 3:**
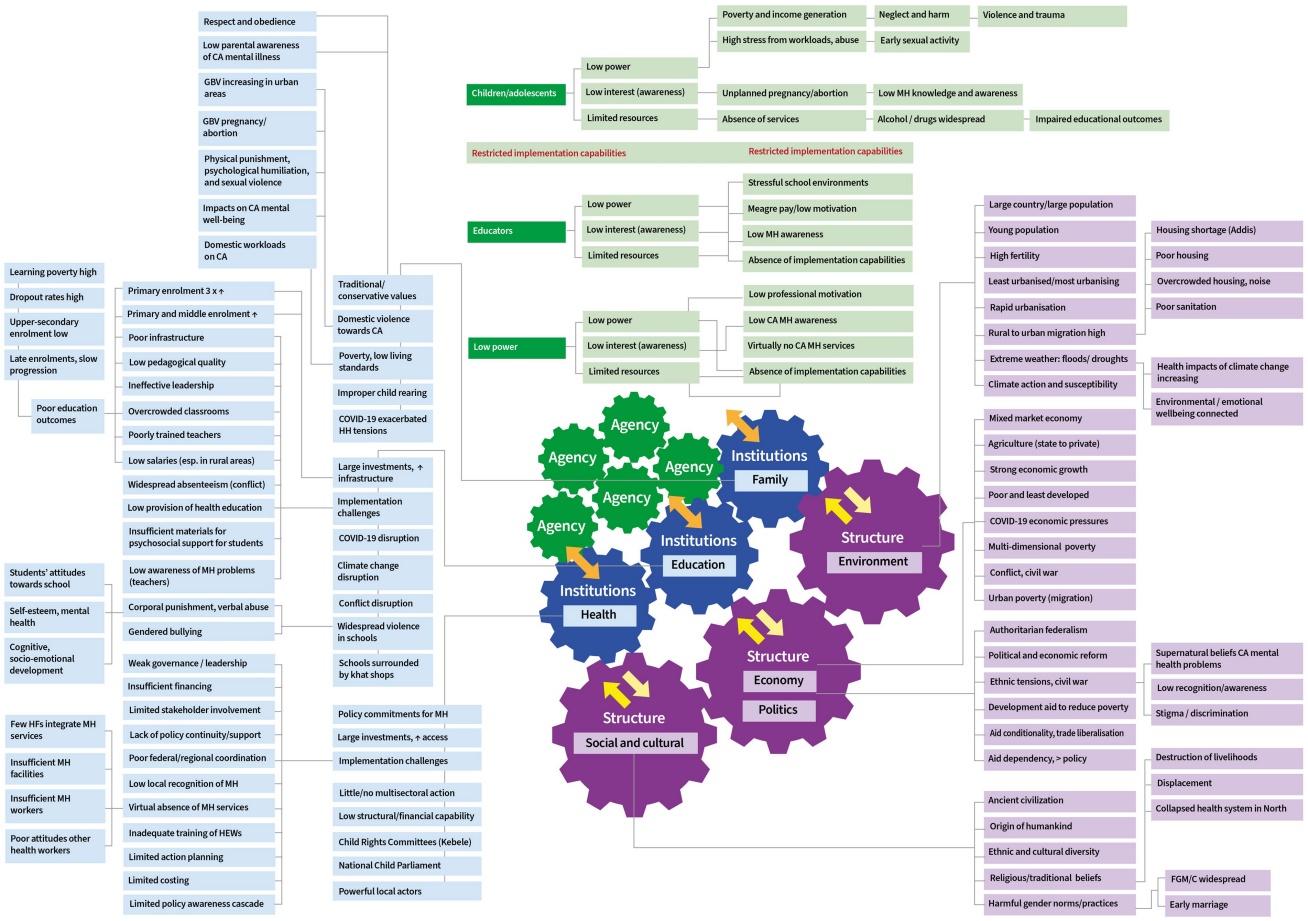
Schema of thematic analysis.

We sought to provide a broad, synthetic view of child and adolescent mental health in Ethiopia. We used PEA to understand multiple, open, complex systems such as the school and family system, and how these interact with statutory systems of education and health, and more widely in global economic and political systems. PEA supports analysis of how these interact to enable and/or constrain the agency of service providers and children and adolescents, and ultimately to locate potential routes for change. Using PEA to examine power dynamics, we identified for instance that local authorities and teachers could be key agents in mental health intervention, yet they face significant resource and training barriers. PEA enabled us to identify interactions between structures, institutions, and individual agency in shaping mental health outcomes; identifying multiple structural features and drivers of mental health problems in the country: globalized urbanization and social fragmentation; and political and economic systems characterized by economic ambition but entrenched poverty, heavy reliance on foreign aid, conflict and civil war. We identified institutional factors affecting exposures to multiple forms of compounding risk, limiting knowledge and acceptability of, and access to, services. Federal resource shortages and poor coordination and dialogue between federal and regional political structures and sectors constrain the collective agencies of service users and providers to protect and promote child and adolescent mental well-being (Figure 3). Key considerations and reflections are presented, below.

One of the fastest urbanizing countries in Africa, Ethiopia is likely to continue to face structural problems such as poor housing, and overcrowding. High rural-to-urban migration may loosen social emotional and financial support networks that help people maintain mental health. The economic climate also poses critical constraints. Ethiopia’s economy is likely to continue to be challenged by migration and relocation, social fragmentation, crime, violence, and youth unemployment (Madoro et al., 2021; Gebreyesus et al., 2023; Mekasha and Tarp, 2021; Central Statistical Agency, 2020). Studies from elsewhere suggest that aid dependency can create challenges, such as high-interest debt repayments, that divert resources from public services like mental health care (Lee et al., 2015). Studies from low and middle income countries (LMICs) confirm how political landscapes, including frequent civil conflicts and governance issues, have significant impacts on citizens’ mental health (Musisi and Kinyanda, 2020; Favara et al., 2022). The conflict in Tigray has also negatively impacted the country’s relationship with western counties and international organizations. Little has been said about the conditionality that comes with the aid and consequences on countries’ development and sovereignty. At this juncture, there is little indication that the local community voice has been considered. This should be seen in a critical light: global actors’ attempts to implement health programs may be unsuccessful without due consideration of local realities and contexts.

The data depicted the profound influence of social norms and cultural perceptions, including tendencies to rely on traditional explanations and treatments, shape attitudes, and influence help-seeking behavior. The study suggests that interventions need to integrate allopathic approaches while respecting cultural contexts, and supports cultural and contextual adaptations of mental health service delivery, and inclusion of religious and spiritual leaders in health literacy activities (Abera et al., 2015; Faregh et al., 2019). The data moreover revealed the profound and multiple exposures to violence for children and adolescents (Chuta et al., 2019; Pankhurst et al., 2016; African Child Policy Forum (ACPF), Save the Children Sweden, 2006a,b; Save the Children Sweden, 2005). The study indicated that parents tend to conceal children with mental illness fearing rejection by society, and that traditional treatments are prioritized as more suitable for mental health problems. Consistent with prior research, stigma remains a major factor deterring treatment for mental health problems in Ethiopia, and health seeking behavior is low (Girma et al., 2022; Hailemariam et al., 2017). International analyses have similarly revealed shortages of stigma reduction strategies for children in LMICs, that mental health problems are commonly understood in combined biological, psychological, and supernatural terms, and that development of interventions should occur directly and indirectly with children and adolescents themselves (Clay et al., 2020; Hartog et al., 2020; Javed et al., 2021; Mascayano et al., 2015; Saraceno et al., 2007).

The analysis identified the potential role of schools and healthcare settings in promoting mental health but also acknowledged significant institutional challenges. The Ethiopian government has identified schools as important setting to implement mental health promotion and prevention programs (Ministry of Education, Ethiopia, 2012). Targeting interventions in schools reflects estimates that 50% of mental health problems start among school-aged children, and that the school community plays a vital role in early intervention for mental health problems involving teachers, school management and parents (Kessler et al., 2005; Hoover and Bostic, 2021). In addition, early identification and treatment can protect children from worsening academic and social functioning. However, there is little information about the implementation of mental health programs and outcomes in school settings. A recent study revealed that the Ethiopian education system is unresponsive to psychosocial and mental health needs of primary school-aged children, for example (Wondie et al., 2011). Devolved administrations operate with limited knowledge transfer, and significant fiscal and resource constraints. The literature indicates that there is substantial regional variation and inequality as a result, and that systematic understanding of region-specific challenges is needed (Yimenu, 2023). Nevertheless, enabling teachers to identify mental health problems is seen as a powerful approach to detect mental problems at early stage, with many teachers in Ethiopia recognizing the importance of school-based mental health intervention including mindfulness and social–emotional learning curricula (Desta et al., 2017; Kerebih et al., 2016). There is a pressing need to enhance the capacity of the school community regarding mental health services. Strengthening collaboration between schools and healthcare services was also recommended.

The Ethiopian government’s tendency to formulate national mental health programs and strategies are recognized as positive steps, but pervasive shortages in dedicated funding and support limit effectiveness. Schools, in turn, lack the needed resources and training to address learners’ mental health issues effectively. Poor coordination between federal and regional political structures is a further challenge. Informant perspectives depicted serious policy-implementation gaps, together with a lack of multisectoral spaces and processes, and chronic resource shortages. Nevertheless, local actor power was raised by several respondents. The study thus supports empowering key actors and agents like teachers, students, and health professionals to become change-makers within the existing structural and institutional frameworks, such as and including child rights forums. Building capacities and fostering supportive relationships could improve overall mental health outcomes. By focusing on entry points, stakeholders can create a supportive environment for child and adolescent mental health. These strategies emphasize the importance of policy advocacy, education, healthcare integration, community engagement, stakeholder collaboration, and data-driven approaches. Based on the PEA, entry points were identified leveraging existing structures, institutions, and agents (Table 3).

**Table 3:** Entry points.

Stakeholder/ sector	Entry point	Objective	Action
Health and education sectors, and institutions	Improve school health programmes with a whole school approach	Build implementation capabilities to integrate mental health into educational environments	Support school-based mental health programs, including mindfulness and social–emotional learning curricula. Train teachers and school staff in mental health awareness and intervention strategies. Support and enable multisectoral spaces and processes, with significant expansion of resources for policy implementation
Policy and governance	Decentralization and local governance to support	Enhance local capacity and autonomy to address mental health issues	Resource and support local governments in developing and implementing context-specific mental health policies and programs. Provide training and significantly expand resources to local implementing officials
Community and cultural engagement	Children, young people and caregivers Community mental health champions	Engage those most directly affected and least represented to ensure relevance Engage community to promote mental health	Development of mental health interventions should occur directly and indirectly with children, adolescents, families and caregivers themselves to ensure relevance and acceptability. Identify community and religious leaders, and influential local actors as mental health champions. Use local platforms for awareness and to reduce stigma.
	Culturally tailored interventions	Ensure mental health interventions are culturally relevant	Develop mental health programs that respect and incorporate local cultural practices and beliefs.
	Community feedback mechanisms	Ensure interventions are responsive to community needs	Establish feedback mechanisms such as community advisory boards and participatory forums to gather input from beneficiaries. Use this feedback to refine and adapt mental health programs
Stakeholder collaboration, including donors and development partners	Multi-sectoral collaboration	Foster a holistic approach to mental health	Establish spaces and processes to coordinate efforts between the ministries of health, education, social affairs, and finance. Promote joint initiatives and significant increase in implementation resource allocation and sharing.
	Foreign aid and development cooperation	Secure funding and technical support	Donors/development partners to align mental health programs with both statutory services and global best practices. Significantly increase harmonised and contextualised implementation support and resources for mental health infrastructure and capacity building.
Monitoring, evaluation, and research	Data collection and research	Build robust evidence base for mental health interventions	Studies to inform policy and practice. Partnerships with stakeholders to evaluate the effectiveness of mental health programs and facilitate uptake of evidence into policy and practice

Addressing these limitations could help create a more responsive system. In considering intervention in schools and healthcare systems, it is crucial to explore feasibility in terms of the social, political and implementation contexts of resource allocation challenges, regional disparities, and political complexities. Given resource scarcity, leveraging existing infrastructure may provide cost-effective mental health services. Schools and healthcare systems can drive adolescent mental health interventions, especially in resource-constrained countries. In schools, this could involve training teachers to identify mental health issues and provide basic psychosocial support, while utilizing school health programs and community-based health workers to extend mental health care to vulnerable populations. In schools, interventions to improve individual well-being, including integrating physical activity into school curricula, as well as those to strengthen social networks, norms, and trust can facilitate coordination and cooperation for mutual benefit (Hunduma et al., 2022; Owens and Hadley, 2023; Li et al., 2024). In healthcare, health extension workers (HEWs) can be trained to recognize mental health concerns and provide basic mental health education, referrals, and support services. Given resource constraints, leveraging low-cost, scalable solutions like peer-to-peer training and community-based health interventions would ensure that services reach vulnerable populations without requiring extensive financial resources. To improve cross-sectoral collaboration, clear communication channels, shared goals, and accountable groups with representatives from education, health, social affairs, and NGOs should be convened to address fragmentation, and develop coordinated approaches aligning efforts. Building cross-sector collaboration requires addressing the communication gaps and creating joint platforms to align mental health goals across sectors. As noted, federal and regional power imbalances complicate policy execution. The allocation of resources is often driven by political priorities, not mental health needs. A transparent budgeting process that prioritizes child and adolescent mental health, alongside other urgent national needs may support to address this. These steps emphasize contextualizing interventions while maximizing available resources. Overcoming policy execution challenges in the current political and resource-constrained environment will require innovative solutions and strong multisectoral collaboration.

### Strengths and limitations

We adopted an approach with a focus on individual behaviors, interactions, and negotiations as they are situated in, reflect, and reinforce the features of multiple, complex, overlapping, open systems. Analysed using PEA, it was possible to identify not only that Ethiopia faces a mental health catastrophe, but potential routes out of it. The study provided a comprehensive analysis of structures, institutions and agents who could influence child and adolescent mental well-being. In this study, we highlighted the complex interactions between structural factors, institutional influences, and individual agency. We identified critical drivers of mental health issues, such as poverty, conflict, and foreign aid dependency, while also exploring social stigma and cultural practices as barriers to mental health care. The study emphasizes the importance of strengthening multisectoral collaboration and empowering local actors to improve mental health outcomes. The study was limited to a small number of informant interviews drawn from statutory, NGO and technical organization practitioner and technical implementation perspectives from key line ministries, domestic and INGOs and academia. While purposive sampling aimed to capture a range of professional and practitioner perspectives, we acknowledge that the small sample size, regional or gender features may have had some influence, limiting the extent to which findings are more generally applicable. Future research that could address these limitations with broader samples from a more regions and gender groups to validate and expand these findings. The literature review enabled detailed documenting of policy and strategy commitments, together with research evidence on prevalence of the burden of child and adolescent mental health problems, remedial and service responses. While small in number, the practitioner, manager and planner perspectives provided complementary insights to the formal literature, on implementation realities and challenges as interconnected phenomenon: whereby different forms of vulnerability and exclusion combine and converge, with amplifying and self-reinforcing effects. These data reflect the value of integrating explicit and tacit knowledge. Further studies should explore the needs and capacities of other stakeholders, critically children and young people, to engage in mental health programs for presentation and treatment.

## Conclusion

Improving child and adolescent mental health in Ethiopia requires a multi-faceted approach that addresses systemic reforms, institutional capacity building, and culturally sensitive interventions. The study reveals significant barriers to the effective implementation of mental health interventions, including power imbalances between federal and regional authorities, financial limitations, and poor coordination. Structural and institutional factors that collectively limit implementers’ capabilities to render health and education services sensitized for mental well-being. The analysis underscored the need for preventive services and robust stakeholder collaboration to foster supportive environments for child and adolescent mental health. We propose leveraging existing systems, such as schools and healthcare settings, as focal points for intervention while addressing barriers. Given the resource constraints, training teachers and healthcare providers in basic mental health interventions is a cost-effective, scalable solution. Integrating community-based health workers into outreach efforts, utilizing existing school health programs, and empowering local actors, such as teachers, health workers, community leaders, and children and adolescents themselves, can enhance capacity building and expand reach. Cross-sector collaboration is critical to overcoming fragmentation, and establishing multistakeholder platforms with shared goals can ensure that mental health is fully integrated into both education and healthcare sectors. To address resource allocation and regional disparities, a strategic, phased approach is required, with targeted efforts to build local capacity and harmonize development partner inputs. Our findings also underscore the importance of aligning policy formulation with context-specific realities, ensuring that mental health strategies are not only formulated but also effectively implemented on the ground.

## Data Availability

The raw data supporting the conclusions of this article will be made available by the authors, without undue reservation.
